# Group education improves self-management of adrenal insufficiency in patients and their relative 

**DOI:** 10.1530/EC-26-0121

**Published:** 2026-07-15

**Authors:** Anna-Karin Åkerman, Charlotte Höybye, Katarina Berinder, Maria Petersson, Tim Spelman, Jeanette Wahlberg, Per Dahlqvist, Sophie Bensing

**Affiliations:** ^1^ Department of Molecular Medicine and Surgery, Karolinska Institutet, Stockholm, Sweden; ^2^ Department of Medicine, Örebro University Hospital, Örebro, Sweden; ^3^ Department of Endocrinology, Karolinska University Hospital, Stockholm, Sweden; ^4^ Department of Clinical Neuroscience, Karolinska Institutet, Stockholm, Sweden; ^5^ School of Medical Sciences, Faculty of Medicine and Health, Örebro University, Örebro, Sweden; ^6^ Department of Public Health and Clinical Medicine, Umeå University, Umeå, Sweden; ^7^ Department of Medicine Solna, Karolinska Institutet, Stockholm, Sweden

**Keywords:** adrenal insufficiency, self-management, relatives, education, group meeting

## Abstract

**Objective:**

Self-management and patient education are cornerstones in managing adrenal insufficiency (AI) to prevent adrenal crisis (AC). Glucocorticoid (GC) education group meetings are increasingly being incorporated into regular healthcare. However, few studies have evaluated its effectiveness.

**Design:**

This is a multicentre pre–post intervention study conducted at four university hospitals in Sweden between 2015 and 2019.

**Methods:**

A total of 254 patients with AI along with 138 relatives were included. Participants attended a GC education group meeting led by an endocrinologist. Detailed guidance was provided on when and how to adjust GC doses to prevent AC together with instructions and supervised practice of hydrocortisone injections. Questionnaires on self-management were completed by both patients and relatives before the session and again 6 months later. In addition, patients also completed two HRQoL instruments.

**Results:**

Patients with AI felt relatively safe and informed about their GC treatment at baseline. However, many lacked adequate knowledge on dose adjustments, including high-risk situations for AC. At follow up, 194 patients (76.4%) and 94 relatives (68.1%) completed the questionnaires. Both patients and relatives showed significant improvements in perceived safety and management of high-risk scenarios for AC. However, some knowledge gaps persisted. The education effort did not result in significant changes in HRQoL.

**Conclusion:**

Structured GC education group meetings enhance the knowledge of patients and their relatives to manage AI safely. We consider this a valuable component of AI care.

**Significance:**

We highlight the value of repeating sick-day rules and using group education to improve self-management in patients with AI and their relatives.

## Introduction

Self-management and patient education are fundamental components in care for patients with chronic diseases, including adrenal insufficiency (AI) (1, 2, 3, 4, 5, 6, 7). In AI, the primary goals of self-management include appropriate adjustment of glucocorticoid (GC) replacement therapy when required and prevention of adrenal crisis (AC), a feared and potentially life-threatening complication (2, 4, 8, 9). Unlike healthy individuals, whose adrenal glands produce and release increased amounts of cortisol during illness or severe stress, patients with AI must manage these situations by increasing their GC replacement therapy (‘stress dosing’) (10, 11, 12). Physicians should provide patients with comprehensive education on the importance of GC dose adjustments during illness, such as fever, vomiting, or diarrhoea, and trauma (5). Patients should carry an emergency card or other information detailing their diagnosis and rules for GC dose adjustment (13). Moreover, guidelines recommend that both patients and their relatives be trained to administer intramuscular hydrocortisone (HC) in emergencies when hospital care is delayed (14). Despite these measures, evidence suggests that knowledge about GC dose adjustments is often insufficient among both patients and healthcare providers, potentially contributing to prolonged illness and the increased mortality risk associated with AI (2, 9, 15, 16, 17, 18, 19, 20, 21). Even severe symptoms do not always lead to a GC dose increase, and many patients and relatives find it difficult to administer the intramuscular HC injection (19, 22, 23). In addition, several studies have reported impaired HRQoL in patients with AI (24, 25, 26, 27, 28), with uncertainty regarding GC dose adjustments potentially contributing to this impairment (29, 30). GC education group meetings are now incorporated into follow-up care in several healthcare settings for patients with AI. However, very few studies have been conducted to evaluate its effectiveness (1, 4) and existing publications primarily focus on patient outcomes, with limited data available on its impact on relatives. The aim of this study was to assess the perception of safety regarding GC treatment and level of knowledge about appropriate GC dose adjustments in patients with AI and their relatives before and after a GC education group meeting. In addition, HRQoL in patients was assessed.

## Methods

### Design

This study was a multicentre pre–post intervention study without a control group.

### Clinical setting

This study was conducted between 2015 and 2019 at four endocrinology units in Swedish university hospitals. During this period, all participating units offered standardised GC education group meetings as part of routine follow-up for patients with AI on GC replacement therapy. These meetings used uniform teaching materials, structure, and time allocation. Each meeting, led by an endocrinologist, lasted approximately three hours and included two short coffee breaks. Groups comprised up to 12 patients, organised by diagnosis: primary adrenal insufficiency (PAI), secondary adrenal insufficiency (SAI), or GC-induced adrenal insufficiency (GIAI). Patients could bring along a relative (a close family member or friend). The meetings featured tailored PowerPoint presentations, one designed for PAI and another for SAI/GIAI, covering the importance of cortisol in maintaining basal metabolism and managing illness and the principles of GC replacement therapy. Detailed guidance was provided on when and how to adjust GC doses, especially to cope with illnesses such as fever, recurrent vomiting, or diarrhoea to prevent AC. Active participation was encouraged through questions and group discussions. At the end of the meeting, a nurse provided oral and video demonstrations on intramuscular HC injection administration. Participants had the opportunity to practice injection technique. Motivated patients were prescribed HC for intramuscular use and received a case containing syringes and needles. GC emergency cards and badges were distributed.

### Participants

Patients with AI scheduled to attend a GC education group meeting as part of their clinical follow-up received study information by postal mail. They were invited to complete questionnaires on self-management and HRQoL both before the meeting and 6 months after. A relative accompanying the patient was also invited, via the patient, to participate as an independent study subject. Both patients and relatives completed the self-management questionnaire at the same time points. Written informed consent was obtained from both patients and relatives.

### Measurements

#### Self-management questionnaire

This study-specific questionnaire, developed by one of the authors (SB), was not formally validated. It was based on a literature review of consensus statements and clinical practice guidelines, including best practice recommendations on the prevention of AC from clinicians experienced in the disease (14, 31).

Key descriptive data collected included year of diagnosis, history of AC, comorbidities, medication, education level, current employment, sick leave, and marital status. Participants rated how safe they felt about the GC replacement overall on a scale from 1 to 10, where 1 indicated ‘not at all’ and 10 indicated ‘completely’. Second, they were asked if they felt that they had received enough information about when and how to adjust their GC doses. Third, they were asked if they thought they knew when and how to adjust the GC dose, both with the following response options: ‘yes, completely’, ‘pretty much’, ‘to some extent’, ‘no, hardly’, ‘not at all’, and ‘no idea’. Finally, the participants were asked how they would adjust their GC dose in the following seven hypothetical situations: *i) a one-hour moderate-intensity workout session, ii) increased stress at work or at home, iii) infection without fever (temp < 38^°^C), iv) infection with fever (temp > 38^°^C), v) an occasional vomiting or diarrhoea, vi) recurrent vomiting or diarrhoea without fever (temp < 38^°^C)*, and *vii) recurrent vomiting or diarrhoea with fever (temp > 38^°^C).*

The definitions of ‘correct’ responses were based on current clinical practice guidelines and expert consensus. However, as high-quality evidence on dose adjustment to prevent AC is limited, recommendations are largely based on clinical experience and best practice statements. The suggested answers in the questionnaire were aligned with these recommendations (14, 31).

We defined the following answers as adequate: *i)* no adjustment, *ii and iii)* no adjustment/raise the daily dose slightly, *iv)* at least double the daily dose, *v)* take one extra tablet of cortisone, *vi and vii)* take one extra tablet of cortisone/inject Solu-Cortef and call for an ambulance.

#### HRQoL questionnaires

The RAND-36 is a licence-free version of Short Form-36 survey, the most common, validated, non-disease-specific instrument assessing subjective health status. It covers eight dimensions of health: physical functioning, role limitations due to physical health, pain, general health, role limitations due to emotional problems, energy/fatigue, emotional well-being, and social functioning. Each dimension is scored on a scale from 1 to 100, with higher scores indicating better HRQoL. AddiQoL is a validated questionnaire specific to Addison’s disease (28, 32), consisting of 30 items that evaluate sub-dimensions, such as emotions, fatigue, symptoms, and miscellaneous aspects. Scores range from 1 to 6 for individual items, with positive HRQoL indicated by higher scores. The total score ranges from 30 to 120, with higher scores reflecting better QoL from a health-related perspective.

### Statistics

Categorical variables are summarised using frequency and percentage. Continuous variables are summarised using mean and standard deviation and/or median and interquartile range (IQR) as appropriate. Comparisons of actions taken in hypothetical situations and AddiQoL- and RAND-36-based outcomes before and after the GC education group meeting were conducted using the Wilcoxon signed-rank test or the McNemar chi-square test, as appropriate, among participants who responded at both time points.

Associations between demographic and diagnosis factors (age, sex, education, diagnosis, and disease duration) and answering no dose adjustment in hypothetical situations after the GC education group meeting were analysed using logistic regression. Effect sizes are reported as odds ratios (ORs) with associated 95% confidence intervals (CIs).

For all analyses, *P* < 0.05 was considered statistically significant. Statistical analysis was performed by Stata, version 18 (StataCorp. 2023. *Stata Statistical Software: Release 18*. USA: StataCorp LLC).

## Results

### Baseline characteristics

A total of 254 patients answered the questionnaires at baseline. The baseline characteristics are presented in Table 1. Most patients had SAI or PAI, with only a fraction having GIAI. Sixty-four percent were female, and the median age at the study start was 54.5 years (IQR: 42–68). Short-acting HC was the most common GC preparation used by 82%, followed by modified release HC in 9.8% of the patients. The daily dose HC equivalent was within recommendations (33). A total of 138 relatives answered the questionnaire at baseline; for socioeconomic variables, see Table 1.

**Table 1:** Baseline characteristics: patients with adrenal insufficiency and relatives.

Characteristic	Patients	Relatives
	*n* = 254	*n* = 138
Gender – *n* (%)		
Female	163 (64.2)	70 (50.7)
Male	91 (35.8)	68 (49.3)
Age – years (IQR)	54.5 (42,68)	62 (50,69)
Marital status – *n* (%)		
Married/cohabitant	178 (70.1)	127 (92)
Single	67 (26.4)	5 (3.6)
Education – *n* (%)		
College/university	129 (50.8)	65 (47.1)
High school	92 (36.2)	54 (32.6)
Elementary school	29 (11.4)	20 (14.5)
Sick leave – *n* (%)		
Partial or 100%	61 (24.0)	4 (2.9)
Diagnosis – *n* (%)		
PAI	119 (46.8)	
SAI	121 (47.6)	
GIAI	14 (5.5)	
Duration of disease – years (IQR)	9 (3, 23)	
GC replacement – *n* (%)		
HC	208 (81.9)	
Modified release HC	25 (9.8)	
Cortisone acetate	13 (5.1)	
Prednisolone	3 (1.2)	
Betamethasone	2 (0.8)	
Daily dose HC equivalent	20 (20, 25)	
Emergency card – *n* (%)	229 (90.2)	
Cortisone badge – *n* (%)	90 (35.4)	
Prescribed GC IV/IM – *n* (%)	126 (49.6)	
Reported ability to inject GC – *n* (%)	84 (33.1)	
Hospital care due to post-diagnosis AC – *n* (%)	110 (43.3)	

PAI, primary adrenal insufficiency; SAI, secondary adrenal insufficiency; GIAI, glucocorticoid-induced adrenal insufficiency; GC, glucocorticoid; HC, hydrocortisone; AC, adrenal crisis; IV, intravenous; IM, intramuscular. Age, duration of disease, and HC equivalent are displayed as median and interquartile range (IQR). The GC replacement dose was recalculated to HC equivalent dose mg as follows: HC dose x 1, cortisone acetate x 0.8; modified release HC x 0.806; prednisolone x 4; betamethasone x 33.

### Self-management at baseline

Patients rated their overall feeling of safety regarding GC treatment with a median score of 8 (IQR: 6–9) on a scale from 1 to 10. The majority (63%) felt they had received enough information on when and how to adjust their GC doses, while 11% did not. When asked if they personally felt they knew when and how to adjust their GC doses, 58% responded affirmatively, while 33% answered ‘to some extent’. A smaller proportion, 7.9%, reported having little to no knowledge in making these adjustments.

In contrast, relatives expressed low confidence across all aspects. They rated their overall feeling of safety regarding GC treatment with a median score of 7 (IQR: 5–9). Only 27% felt they had received enough information on dose adjustments. When asked if they knew when and how their relative should adjust their GC dose, 32% responded yes, while 42% answered ‘to some extent’. Notably, 25% indicated they had little or no understanding of how to manage GC adjustments.

### Actions in hypothetical situations – at baseline

**Figure 1:**
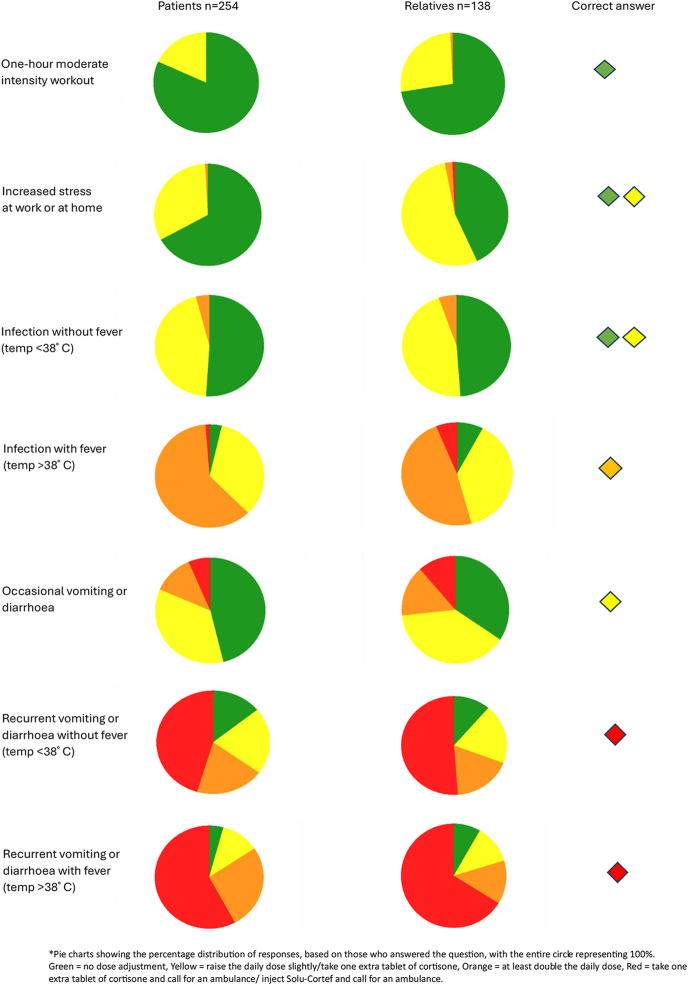
Patient- and relative-reported actions in hypothetical situations, before the GC education group meeting.

For an overview of how patients and relatives suggested adjusting the GC dose in the seven hypothetical situations, see Fig. 1, and for detailed information, see Supplementary Table 1 (see section on Supplementary materials given at the end of the article).

Overall, both patients and relatives seemed to have a good understanding of how to manage GC dosing in low-risk situations for AC. For infection without fever (temp < 38^°^C), 91% (*n* = 232) of patients and 81% (*n* = 112) relatives provided the correct response.

For infections with fever (temp > 38^°^C), 60% (*n* = 153) of patients and 46% (*n* = 63) of relatives recommended the correct adjustment. In cases of occasional vomiting or diarrhoea, there was considerable uncertainty about the appropriate course of action, with a wide range of suggested measures.

In the two highest risk situations for AC, recurrent vomiting or diarrhoea with or without fever, most participants suggested some form of dose increase, while only 54% (*n* = 137) and 43% (*n* = 109) of patients and 64% (*n* = 88) and 49% (*n* = 68) of relatives provided a correct answer. A small proportion of patients and relatives even suggested no dose adjustment.

### Follow-up 6 months after the GC education group meeting

A total of 194 patients (76%) and 94 relatives (68%) completed the questionnaires both before the GC education group meeting and at follow-up. Socioeconomic characteristics and type of AI were compared between the baseline cohorts and participants who completed the questionnaires at both time points. Predictors of patient dropout were younger age (OR: 0.97; 95% CI: 0.95–0.99; *P* < 0.001) and higher education: high school (OR: 13.55; 95% CI: 1.76–104.37; *P* = 0.012) and college/university (OR: 7.76; 95% CI: 1.01–59.58; *P* = 0.049) compared with elementary school. Patients unable to inject Solu-Cortef were more likely to drop out compared with those who stated they could (OR: 0.42; 95% CI: 0.20–0.86; *P* = 0.018). No significant predictors of dropout were observed among relatives.

Among patients who also completed the follow-up, no change in daily HC equivalent dose was observed (*P* = 0.732). However, the proportion of patients stating they knew how to inject Solu-Cortef significantly increased from 38% (*n* = 73) to 81% (*n* = 158) (*P* < 0.001). The proportion carrying an emergency card rose from 89 to 97% (*P* = 0.001), and those equipped with a cortisone badge increased from 37 to 62% (*P* < 0.001).

### Confidence in self-management at follow-up

Patients’ overall feeling of safety regarding the GC treatment significantly increased from a median of 8 (IQR: 7–9) before to 9 (IQR: 7–10) after the GC education group meeting (*P* < 0.001). The proportion of patients who felt they had received enough information on when and how to adjust the GC dose increased from 67 to 83%, while those reporting receiving insufficient information decreased from 11 to 1.0% (*P* < 0.001). Similarly, the proportion of patients reporting that they knew when and how to adjust the GC dose rose from 60 to 79%, with insufficient knowledge declining from 7.3 to 1.0% (*P* < 0.001).

Relatives’ overall feeling of safety regarding their relatives’ GC treatment significantly improved, rising from a median of 7 (IQR: 5–9) to 8 (IQR: 7–9) after the GC education group meeting (*P* < 0.001). The proportion reporting to have received enough information on when and how to adjust the GC dose increased from 23 to 76%, while that reporting insufficient information decreased from 38 to 3.2% (*P* = 0.016). The relatives’ self-assessed sufficient knowledge of when and how their relative should adjust the GC dose increased from 30 to 68%, while the proportion reporting insufficient knowledge dropped from 26 to 5.3% at follow-up (*P* = 0.048).

### Actions in hypothetical situations at follow-up

We detected significant changes in both patient- and relative-reported actions across all seven hypothetical scenarios; see Tables 2 and 3.

**Table 2:** Patient-reported actions in hypothetical situations, before and after the GC education group meeting.

Hypothetical situation	Action	Patients *n* = 194		*P*-value
		Before *n* (%)	After *n* (%)	
One hour moderate-intensity workout	No dose adjustment	159 (82.0)	153 (78.9)	<0.001
	Raise the daily dose slightly	20 (10.3)	30 (15.5)	
	Take one extra tablet of cortisone	9 (4.6)	10 (0.5)	
	At least double the daily dose	0 (0.0)	0 (0.0)	
	Take one extra tablet of cortisone and call for an ambulance	0 (0.0)	0 (0.0)	
	Inject Solu-Cortef and call for an ambulance	0 (0.0)	0 (0.0)	
	Not reported or N/A	6 (3.0)	1 (0.5)	
Increased stress at work or at home	No dose adjustment	131 (67.5)	111 (57.2)	<0.001
	Raise the daily dose slightly	43 (22.2)	65 (33.5)	
	Take one extra tablet of cortisone	12 (6.2)	16 (8.3)	
	At least double the daily dose	2 (1.0)	0 (0.0)	
	Take one extra tablet of cortisone and call for an ambulance	0 (0.0)	0 (0.0)	
	Inject Solu-Cortef and call for an ambulance	0 (0.0)	0 (0.0)	
	Not reported or N/A	6 (3.1)	2 (1.0)	
Infection without fever (temp < 38^°^C)	No dose adjustment	93 (47.9)	95 (49.0)	<0.001
	Raise the daily dose slightly	84 (43.3)	72 (37.1)	
	Take one extra tablet of cortisone	7 (3.6)	13 (6.7)	
	At least double the daily dose	7 (3.6)	12 (6.2)	
	Take one extra tablet of cortisone and call for an ambulance	0 (0.0)	1 (0.5)	
	Inject Solu-Cortef and call for an ambulance	0 (0.0)	0 (0.0)	
	Not reported or N/A	3 (1.6)	1 (0.5)	
Infection with fever (temp > 38^°^C)	No dose adjustment	7 (3.6)	6 (3.1)	<0.001
	Raise the daily dose slightly	48 (24.7)	32 (16.5)	
	Take one extra tablet of cortisone	16 (8.3)	19 (9.8)	
	At least double the daily dose	116 (59.8)	132 (68.0)	
	Take one extra tablet of cortisone and call for an ambulance	3 (1.6)	2 (1.0)	
	Inject Solu-Cortef and call for an ambulance	0 (0.0)	2 (1.0)	
	Not reported or N/A	4 (2.1)	1 (0.5)	
Occasional vomiting or diarrhoea	No dose adjustment	91 (46.9)	66 (34.0)	<0.001
	Raise the daily dose slightly	37 (19.1)	42 (21.7)	
	Take one extra tablet of cortisone	25 (12.9)	44 (22.7)	
	At least double the daily dose	22 (11.3)	25 (12.9)	
	Take one extra tablet of cortisone and call for an ambulance	10 (5.2)	8 (4.1)	
	Inject Solu-Cortef and call for an ambulance	5 (2.6)	5 (2.6)	
	Not reported or N/A	4 (2.1)	4 (2.1)	
Recurrent vomiting or diarrhoea without fever (temp < 38^°^C)	No dose adjustment	27 (13.9)	13 (6.7)	<0.001
	Raise the daily dose slightly	25 (12.9)	22 (11.3)	
	Take one extra tablet of cortisone	13 (6.7)	21 (10.8)	
	At least double the daily dose	39 (20.1)	36 (18.6)	
	Take one extra tablet of cortisone and call for an ambulance	49 (25.3)	45 (23.2)	
	Inject Solu-Cortef and call for an ambulance	32 (16.5)	55 (28.4)	
	Not reported or N/A	9 (4.6)	2 (1.0)	
Recurrent vomiting or diarrhoea with fever (temp > 38^°^C)	No dose adjustment	7 (3.6)	3 (1.6)	<0.001
	Raise the daily dose slightly	10 (5.2)	5 (2.6)	
	Take one extra tablet of cortisone	9 (4.6)	10 (5.2)	
	At least double the daily dose	54 (27.8)	35 (18.0)	
	Take one extra tablet of cortisone and call for an ambulance	62 (32.0)	50 (25.8)	
	Inject Solu-Cortef and call for an ambulance	40 (20.6)	88 (45.4)	
	Not reported or N/A	12 (6.2)	3 (1.6)	

**Table 3:** Relative-reported actions in hypothetical situations, before and after the GC education group meeting.

Hypothetical situation	Action	Relatives *n* = 94		*P*-value
		Before *n* (%)	After *n* (%)	
One hour moderate-intensity workout	No dose adjustment	66 (70.2)	60 (63.8)	<0.001
	Raise the daily dose slightly	14 (14.9)	16 (17.0)	
	Take one extra tablet of cortisone	9 (9.6)	16 (17.0)	
	At least double the daily dose	0 (0.0)	2 (2.1)	
	Take one extra tablet of cortisone and call for an ambulance	0 (0.0)	0 (0.0)	
	Inject Solu-Cortef and call for an ambulance	0 (0.0)	0 (0.0)	
	Not reported or N/A	5 (5.3)	0 (0.0)	
Increased stress at work or at home	No dose adjustment	46 (48.9)	37 (39.4)	<0.001
	Raise the daily dose slightly	30 (31.9)	40 (42.6)	
	Take one extra tablet of cortisone	11 (11.7)	16 (17.0)	
	At least double the daily dose	2 (2.1)	1 (1.1)	
	Take one extra tablet of cortisone and call for an ambulance	0 (0.0)	0 (0.0)	
	Inject Solu-Cortef and call for an ambulance	0 (0.0)	0 (0.0)	
	Not reported or N/A	5 (5.3)	0 (0.0)	
Infection without fever (temp < 38^°^C)	No dose adjustment	49 (52.1)	35 (37.2)	0.005
	Raise the daily dose slightly	27 (28.7)	43 (45.7)	
	Take one extra tablet of cortisone	8 (8.5)	10 (10.6)	
	At least double the daily dose	5 (5.3)	5 (5.3)	
	Take one extra tablet of cortisone and call for an ambulance	0 (0.0)	1 (1.1)	
	Inject Solu-Cortef and call for an ambulance	0 (0.0)	0 (0.0)	
	Not reported or N/A	5 (5.3)	0 (0.0)	
Infection with fever (temp > 38^°^C)	No dose adjustment	9 (9.6)	2 (2.1)	0.009
	Raise the daily dose slightly	25 (26.6)	16 (17.0)	
	Take one extra tablet of cortisone	7 (7.5)	10 (10.6)	
	At least double the daily dose	42 (44.7)	57 (60.6)	
	Take one extra tablet of cortisone and call for an ambulance	5 (5.3)	7 (7.5)	
	Inject Solu-Cortef and call for an ambulance	1 (1.1)	2 (2.1)	
	Not reported or N/A	5 (5.3)	0 (0.0)	
Occasional vomiting or diarrhoea	No dose adjustment	35 (37.2)	24 (25.5)	<0.001
	Raise the daily dose slightly	14 (14.9)	15 (16.0)	
	Take one extra tablet of cortisone	15 (16.0)	30 (31.9)	
	At least double the daily dose	15 (16.0)	15 (16.0)	
	Take one extra tablet of cortisone and call for an ambulance	6 (6.4)	8 (8.5)	
	Inject Solu-Cortef and call for an ambulance	4 (4.3)	2 (2.1)	
	Not reported or N/A	5 (5.3)	0 (0.0)	
Recurrent vomiting or diarrhoea without fever (temp < 38^°^C)	No dose adjustment	12 (12.8)	5 (5.3)	<0.001
	Raise the daily dose slightly	11 (11.7)	7 (7.5)	
	Take one extra tablet of cortisone	6 (6.4)	8 (8.5)	
	At least double the daily dose	20 (21.3)	14 (14.9)	
	Take one extra tablet of cortisone and call for an ambulance	23 (24.5)	27 (28.7)	
	Inject Solu-Cortef and call for an ambulance	18 (19.2)	33 (35.1)	
	Not reported or N/A	4 (4.3)	0 (0.0)	
Recurrent vomiting or diarrhoea with fever (temp > 38^°^C)	No dose adjustment	8 (8.5)	1 (1.1)	<0.001
	Raise the daily dose slightly	8 (8.5)	1 (1.1)	
	Take one extra tablet of cortisone	3 (3.2)	2 (2.1)	
	At least double the daily dose	16 (17.0)	8 (8.5)	
	Take one extra tablet of cortisone and call for an ambulance	32 (34.0)	28 (29.8)	
	Inject Solu-Cortef and call for an ambulance	24 (25.5)	54 (57.5)	
	Not reported or N/A	3 (3.2)	0 (0.0)	

In low-risk scenarios for AC, the correct actions were still suggested by most patients and relatives. In cases of infection with fever (temp > 38^°^C), more patients (68%, *n* = 132) and relatives (61%, *n* = 57) proposed the correct adjustment. In cases of occasional vomiting or diarrhoea, there was still significant uncertainty regarding the appropriate course of action. However, more individuals deviated from not adjusting the dose, to take an additional tablet of cortisone.

**Figure 2:**
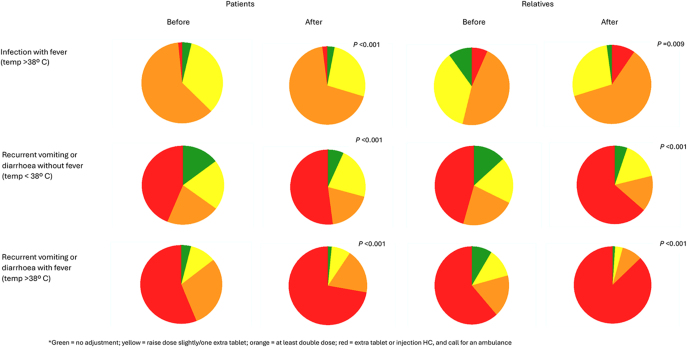
Patient- and relative-reported actions in hypothetical situations, before and after the GC education group meeting.

In the two highest risk situations for AC, recurrent vomiting or diarrhoea with or without fever, now more patients and relatives correctly recommended the desired answer. However, some of the participants, especially patients, still suggested only administering additional oral GC and not seeking medical attention. Figure 2 graphically presents the actions suggested by patients and relatives in the three scenarios with the highest risk of AC.

Older age was associated with a higher likelihood of reporting no dose adjustment in the event of occasional vomiting or diarrhoea, and the corresponding ORs were 1.03 (95% CI: 1.01–1.06; *P* = 0.002) in patients and 1.08 (95% CI: 1.02–1.15; *P* = 0.005) in relatives. And the same association with older age and no dose adjustment were noticed for situations with recurrent vomiting or diarrhoea without fever, both among patients (OR: 1.08; 95% CI: 1.02–1.14; *P* = 0.009) and relatives (OR: 1.24; 95% CI: 1.03–1.48; *P* = 0.020).

Having a diagnosis of SAI was associated with a greater likelihood of reporting no dose adjustment in cases of recurrent vomiting or diarrhoea without fever (OR: 10.87; 95% CI: 1.37–86.10; *P* = 0.024). Female patients were less likely than male patients to report no dose adjustment in response to occasional vomiting or diarrhoea (OR: 0.46; 95% CI: 0.25–0.85; *P* = 0.013).

### Health-related quality of life

Health-related quality of life was assessed using the AddiQoL questionnaire and the RAND-36 instrument. Legitimate AddiQoL values were obtained from 213 of 254 patients before and 169 of 194 patients after 6 months. Analysis among participants completing the questionnaire at both times showed no significant changes following the GC group education meeting, with median scores of 78 (IQR: 69–87) before and 80 (IQR: 71–87) after (*P* = 0.650). For RAND-36, legitimate values were available for 243 patients before and 183 after, and among those who responded at both time points, no significant changes were observed in any dimensions.

## Discussion

This study found that although patients with AI felt relatively safe and informed about GC treatment at baseline, many lacked adequate knowledge on dose adjustments, including high-risk situations for AC. Following a GC education group meeting, both patients and relatives showed significant improvements in perceived safety and management of high-risk scenarios. However, some knowledge gaps persisted.

Cortisol plays a crucial role in regulating blood pressure and fluid balance. During infections, when the body loses fluids through sweating, vomiting, or diarrhoea, maintaining stable blood volume and blood pressure becomes even more critical. In addition, cortisol is important to modulate the inflammatory response. Consequently, in patients with AI, it is standard practice to at least double the GC dose in case of fever (34). Despite this, about one-third of the patients in this study reported that they would not double the daily GC dose during fever, prior to participating in the GC education group meeting, which is concerning. The GC education group meeting led to an improvement, with more patients responding correctly in this situation. However, a significant proportion still indicated that they would not double their GC dose. Another cause of concern was the number of patients who stated they would not seek medical care in the event of recurrent vomiting or diarrhoea, which has also been observed in a German study by Schöfl *et al.* (19). Several studies have clearly linked the increased mortality risk in AI to infections, with gastrointestinal infections identified as a major trigger for AC (33, 35, 36). In these cases, immediate administration of intravenous or intramuscular HC, along with intravenous saline solution, is crucial to prevent fatalities (33).

There may be several potential reasons why patients do not act according to recommendations in situations with high risk of AC. It cannot completely be assumed that healthcare professionals consistently convey the importance of GC stress dosing during clinical visits. If this critical information is not clearly communicated, it represents a shortcoming in the medical management and support of patients with AI. To ensure proper patient education, healthcare providers themselves must receive adequate training and possess sufficient knowledge about stress dosing principles and the risks associated with AC. Encouragingly, most patients were equipped with an emergency card already prior to the GC education group meeting, which should facilitate appropriate emergency medical care; however, only a minority could administer Solu-Cortef themselves.

In this study, older age and a diagnosis of SAI were identified as predictors for not adjusting the GC dose in cases of recurrent vomiting or diarrhoea without fever. Other researchers have also reported a negative correlation between age and knowledge, indicative of a better knowledge in the younger patients (21). Some patients with SAI retain partial adrenocorticotropic hormone (ACTH) function, which may buffer low- to moderate-risk situations, leading to a reduced perceived need for GC dose escalation or seeking medical care. In addition, the preserved aldosterone production in patients with SAI offers better regulation of sodium balance and cardiovascular function compared with those with PAI, potentially masking the severity of AI symptoms. Moreover, individuals with SAI caused by hypothalamic–pituitary damage, for example, after surgery, radiotherapy, or trauma, may experience cognitive impairments, limiting their ability to recognise or comprehend the need for stress dosing. In contrast, patients with PAI often experience AI as a central and more immediately apparent condition, making it a shared clinical priority for both patients and healthcare professionals. Patients with SAI, on the other hand, frequently present with multiple hormonal deficiencies that compete for attention during medical consultations. As a result, communication regarding GC stress dosing may be less explicit, and patient comprehension may be more limited. Nevertheless, AC remains a significant contributor to increased mortality in SAI (37), and it is essential that both healthcare providers and patients understand the importance of stress dosing with GC in SAI (36). Another contributing factor for not actively making dose adjustments could be the long intervals between high-risk episodes, during which patients may forget the appropriate actions to take or concerns about possible adverse effects of GC treatment (38).

It is perhaps not surprising that relatives feel less informed and more uncertain about GC dosing than patients at baseline. Traditionally, education on GC stress dosing has been provided during doctor’s appointments, usually with only the patient present. However, following the GC education group meeting, there were significant improvements in how safe and informed the relatives felt. Interestingly, higher percentages of relatives than patients then correctly answered questions about how to act in situations with high risk of AC, recurrent vomiting or diarrhoea. Relatives can play a crucial role in supporting patients and helping them make the right decisions in high-risk situations for AC. They can assist the patient taking additional GC, help administer intramuscular HC, and call an ambulance if needed. It is also a benefit that relatives are well informed about the patient’s condition, so they can advocate for them and ensure the timely administration of parenteral HC and saline when required. A patient with AI who becomes severely ill may not respond appropriately and may be unable to clearly express their healthcare needs, making the role of a knowledgeable relative even more vital.

Following the GC education group meeting, both patients and relatives showed a greater tendency to increase the oral GC dose slightly before moderate-intensity workout, during periods of increased mental stress, or in the presence of infection without fever. Current guidelines state that GC dose adjustments may be necessary during intense physical activity, infection without fever, or increased mental stress, provided the patient perceives that such adjustments alleviate symptoms of AI and promote a faster recovery (39). An inverse relationship between the frequency of AC and the self-reported frequency of dose adjustments has been documented (40). Therefore, potential benefits of small oral GC dose adjustments may be obtained, enabling patients to recognise that they can actively influence their health.

Patient-reported HRQoL remained unchanged following the GC education group meeting, suggesting no impact or insufficient follow-up time to detect any meaningful impact on RAND-36 scores in line with previous studies on AI (41). Educational levels among both patients and relatives appeared higher than those reported in a Swedish nationwide study on socioeconomic factors in individuals with autoimmune Addison’s disease (42), likely reflecting the recruitment setting at university hospitals, where individuals with higher educational attainment may be overrepresented. The odds of dropout among patients were predicted by younger age, higher educational level, and inability to inject Solu-Cortef. This may suggest that these patients felt sufficiently informed, did not prioritise follow-up, or perceived less need for continued support.

In conclusion, structured GC education group meetings clearly strengthen the ability of patients with AI and their relatives to manage stress dosing and thereby support safe disease management. However, a minority of participants still failed to fully grasp or retain the information on dose adjustments. Identifying the reasons for this and providing targeted support remain crucial. These findings highlight the value of including GC education group meetings into the follow-up of AI, while also emphasising the potential benefit of re-education to ensure sustained knowledge and safe long-term self-management.

## Supplementary materials



## Declaration of interest

The authors declare that there is no conflict of interest that could be perceived as prejudicing the impartiality of the work reported.

## Funding

This work was supported by the Regional Agreement on Medical Training and Clinical Research between Stockholm County Council (ALF) and Karolinska Institutet (SB).

## Ethics

This study was approved by the Regional Ethical Review Board in Stockholm (2008/296-31/12; 2014/1577-31/12).

## References

[1] Repping-Wuts HJ, Stikkelbroeck NM, Noordzij A, et al. A glucocorticoid education group meeting: an effective strategy for improving self-management to prevent adrenal crisis. Eur J Endocrinol 2013 169 17–22. (10.1530/eje-12-1094) 23636446

[2] Pazderska A & Pearce SH. Adrenal insufficiency – recognition and management. Clin Med 2017 17 258–262. (10.7861/clinmedicine.17-3-258) PMC6297573 28572228

[3] Bouziane T, Belmahi N, Salhi H, et al. Knowledge and attitude of patients with adrenal insufficiency. Ann Afr Med 2020 19 252–257. (10.4103/aam.aam_63_19) 33243948 PMC8015952

[4] Burger-Stritt S, Eff A, Quinkler M, et al. Standardised patient education in adrenal insufficiency: a prospective multi-centre evaluation. Eur J Endocrinol 2020 183 119–127. (10.1530/eje-20-0181) 32580144

[5] Ahmet A, Gupta A, Malcolm J, et al. Approach to the patient: preventing adrenal crisis through patient and clinician education. J Clin Endocrinol Metab 2023 108 1797–1805. (10.1210/clinem/dgad003) 36630291

[6] Kampmeyer D, Haas CS, Moenig H, et al. Self-management in adrenal insufficiency – towards a better understanding. Endocr J 2017 64 379–385. (10.1507/endocrj.ej16-0429) 28190868

[7] Albarel F, Pellegrini I, Rahabi H, et al. Evaluation of an individualized education program in pituitary diseases: a pilot study. Eur J Endocrinol 2020 183 551–559. (10.1530/eje-20-0652) 33055299

[8] Shepherd LM, Schmidtke KA, Hazlehurst JM, et al. Interventions for the prevention of adrenal crisis in adults with primary adrenal insufficiency: a systematic review. Eur J Endocrinol 2022 187 S1–S20. (10.1530/eje-21-1248) 35536876 PMC9175553

[9] Puar TH, Stikkelbroeck NM, Smans LC, et al. Adrenal crisis: still a deadly event in the 21st century. Am J Med 2016 129 339.e1–339.e9. (10.1016/j.amjmed.2015.08.021) 26363354

[10] Hahner S, Ross RJ, Arlt W, et al. Adrenal insufficiency. Nat Rev Dis Primers 2021 7 19. (10.1038/s41572-021-00252-7) 33707469

[11] Russell GM, Kalafatakis K & Lightman SL. The importance of biological oscillators for hypothalamic–pituitary–adrenal activity and tissue glucocorticoid response: coordinating stress and neurobehavioural adaptation. J Neuroendocrinol 2015 27 378–388. (10.1111/jne.12247) 25494867 PMC4539599

[12] Bensing S, Hulting AL, Husebye ES, et al. Management of endocrine disease: epidemiology, quality of life and complications of primary adrenal insufficiency: a review. Eur J Endocrinol 2016 175 R107–R116. (10.1530/eje-15-1242) 27068688

[13] Dahlqvist P, Bensing S, Ekwall O, et al. [A national medical emergency card for adrenal insufficiency. A new warning card for better management and patient safety]. Lakartidningen 2011 108 2226–2227. 22165183

[14] Bornstein SR, Allolio B, Arlt W, et al. Diagnosis and treatment of primary adrenal insufficiency: an Endocrine Society Clinical Practice Guideline. J Clin Endocrinol Metab 2016 101 364–389. (10.1210/jc.2015-1710) 26760044 PMC4880116

[15] Flemming TG & Kristensen LO. Quality of self-care in patients on replacement therapy with hydrocortisone. J Intern Med 1999 246 497–501. (10.1046/j.1365-2796.1999.00538.x) 10583719

[16] van Eck JP, Gobbens RJ, Beukers J, et al. Much to be desired in self-management of patients with adrenal insufficiency. Int J Nurs Pract 2016 22 61–69. (10.1111/ijn.12368) 25353148

[17] Claessen K, Andela CD, Biermasz NR, et al. Clinical unmet needs in the treatment of adrenal crisis: importance of the patient’s perspective. Front Endocrinol 2021 12 701365. (10.3389/fendo.2021.701365) PMC8329717 34354671

[18] van der Meij NT, van Leeuwaarde RS, Vervoort SC, et al. Self-management support in patients with adrenal insufficiency. Clin Endocrinol 2016 85 652–659. (10.1111/cen.13083) 27063934

[19] Schöfl C, Mayr B, Maison N, et al. Daily adjustment of glucocorticoids by patients with adrenal insufficiency. Clin Endocrinol 2019 91 256–262. (10.1111/cen.14004) 31050815

[20] White KG. A retrospective analysis of adrenal crisis in steroid-dependent patients: causes, frequency and outcomes. BMC Endocr Disord 2019 19 129. (10.1186/s12902-019-0459-z) 31791297 PMC6889201

[21] Ross IL, Llahana S, Anderson MM, et al. Identifying knowledge gaps in individuals with primary adrenal insufficiency: a critical step in preventing adrenal crisis. Clin Endocrinol 2025 103 659–668. (10.1111/cen.70006) PMC12492782 40696758

[22] Llahana S, Anthony J, Sarafoglou K, et al. Patient and caregiver experiences with hydrocortisone injections in adrenal crisis: a mixed-methods cross-sectional study. Front Endocrinol 2025 16 1544502. (10.3389/fendo.2025.1544502) PMC12053486 40331138

[23] Hover WJ, Krein AD, Kallet J, et al. People with adrenal insufficiency who are in adrenal crisis are frequently unable to self-administer rescue injections. Endocr Pract 2025 31 625–630. (10.1016/j.eprac.2025.02.017) 40043845

[24] Touraine P, Chenuc G & Colin C. Self-perceived health status of patients with adrenal insufficiency receiving glucocorticoid replacement therapy – french data from a worldwide patient survey. Ann Endocrinol 2015 76 9–12. (10.1016/j.ando.2014.09.003) 25573224

[25] Li D, Genere N, Behnken E, et al. Determinants of self-reported health outcomes in adrenal insufficiency: a multisite survey study. J Clin Endocrinol Metab 2021 106 e1408–e1419. (10.1210/clinem/dgaa668) 32995875 PMC7947833

[26] van der Valk ES, Smans LC, Hofstetter H, et al. Decreased physical activity, reduced QoL and presence of debilitating fatigue in patients with Addison’s disease. Clin Endocrinol 2016 85 354–360. (10.1111/cen.13059) 26953557

[27] De Bucy C, Guignat L, Niati T, et al. Health-related quality of life of patients with hypothalamic–pituitary–adrenal axis dysregulations: a cohort study. Eur J Endocrinol 2017 177 1–8. (10.1530/eje-17-0048) 28404594

[28] Oksnes M, Bensing S, Hulting AL, et al. Quality of life in European patients with Addison’s disease: validity of the disease-specific questionnaire AddiQoL. J Clin Endocrinol Metab 2012 97 568–576. (10.1210/jc.2011-1901) 22090270

[29] Tiemensma J, Andela CD, Pereira AM, et al. Patients with adrenal insufficiency hate their medication: concerns and stronger beliefs about the necessity of hydrocortisone intake are associated with more negative illness perceptions. J Clin Endocrinol Metab 2014 99 3668–3676. (10.1210/jc.2014-1527) 25226291

[30] Meyer G, Koch M, Herrmann E, et al. Longitudinal AddiQoL scores may identify higher risk for adrenal crises in Addison’s disease. Endocrine 2018 60 355–361. (10.1007/s12020-017-1513-0) 29388043

[31] Husebye ES, Allolio B, Arlt W, et al. Consensus statement on the diagnosis, treatment and follow-up of patients with primary adrenal insufficiency. J Intern Med 2014 275 104–115. (10.1111/joim.12162) 24330030

[32] Lovas K, Curran S, Oksnes M, et al. Development of a disease-specific quality of life questionnaire in Addison’s disease. J Clin Endocrinol Metab 2010 95 545–551. (10.1210/jc.2009-1711) 20016050

[33] Husebye ES, Pearce SH, Krone NP, et al. Adrenal insufficiency. Lancet 2021 397 613–629. (10.1016/s0140-6736(21)00136-7) 33484633

[34] Nowotny H, Ahmed SF, Bensing S, et al. Therapy options for adrenal insufficiency and recommendations for the management of adrenal crisis. Endocrine 2021 71 586–594. (10.1007/s12020-021-02649-6) 33661460 PMC7929907

[35] White K & Arlt W. Adrenal crisis in treated Addison’s disease: a predictable but under-managed event. Eur J Endocrinol 2010 162 115–120. (10.1530/eje-09-0559) 19776201

[36] Hahner S, Spinnler C, Fassnacht M, et al. High incidence of adrenal crisis in educated patients with chronic adrenal insufficiency: a prospective study. J Clin Endocrinol Metab 2015 100 407–416. (10.1210/jc.2014-3191) 25419882

[37] Burman P, Mattsson AF, Johannsson G, et al. Deaths among adult patients with hypopituitarism: hypocortisolism during acute stress, and de novo malignant brain tumors contribute to an increased mortality. J Clin Endocrinol Metab 2013 98 1466–1475. (10.1210/jc.2012-4059) 23457412

[38] Chapman SC, Llahana S, Carroll P, et al. Glucocorticoid therapy for adrenal insufficiency: nonadherence, concerns and dissatisfaction with information. Clin Endocrinol 2016 84 664–671. (10.1111/cen.12991) 26641418

[39] Beun JG, Burman P, Kämpe O, et al. Doctors, teach your adrenal insufficiency patients well: provide them with a European Emergency Card!. Endocr Connect 2023 12 e220345. (10.1530/ec-22-0345) 36327148 PMC9782421

[40] Chifu I, Burger-Stritt S, Schrader A, et al. Predisposing factors for adrenal crisis in chronic adrenal insufficiency: a case-control study. Eur J Endocrinol 2023 189 537–545. (10.1093/ejendo/lvad149) 38006230

[41] Hahner S, Loeffler M, Fassnacht M, et al. Impaired subjective health status in 256 patients with adrenal insufficiency on standard therapy based on cross-sectional analysis. J Clin Endocrinol Metab 2007 92 3912–3922. (10.1210/jc.2007-0685) 17684047

[42] Stergianos S, Everhov ÅH, Söderling J, et al. Income and work loss in patients with Addison’s disease: a nationwide population-based study. Eur J Endocrinol 2025 192 170–179. (10.1093/ejendo/lvaf022) 39980335

