# Supplementary material for: Group education improves self-management of adrenal insufficiency in patients and their relative
Source: Endocr Connect. 2026 Jul 15;15(7):e260121. doi: 10.1530/EC-26-0121 (PMC13386157; doi:10.1530/EC-26-0121)
Supplement: Supplementary file 1 [file EC-26-0121_supplementary_table.pdf]

Supplementary table 1: Patient and relative-reported actions in hypothetical situations, before the GC education group meeting.

Hypothetical situation	Action	Patients n=254 n (%)	Relatives n=138 n (%)
One hour moderate-intensity workout	No dose adjustment	203 (79.9)	95 (68.8)
	Raise the daily dose slightly	27 (10.6)	22 (15.9)
	Take one extra tablet of cortisone	18 (7.1)	13 (9.4)
	At least double the daily dose	0 (0.0)	1 (0.7)
	Take one extra tablet of cortisone and call for an ambulance	0 (0.0)	0 (0.0)
	Inject Solu-Cortef and call for an ambulance	0 (0.0)	0 (0.0)
	Not reported or N/A	6 (2.4)	7 (5.1)
Increased stress at work or at home	No dose adjustment	166 (65.4)	57 (41.3)
	Raise the daily dose slightly	62 (24.4)	52 (37.7)
	Take one extra tablet of cortisone	18 (7.1)	19 (13.8)
	At least double the daily dose	2 (0.8)	3 (2.2)
	Take one extra tablet of cortisone and call for an ambulance	0 (0.0)	0 (0.0)
	Inject Solu-Cortef and call for an ambulance	0 (0.0)	1 (0.7)
	Not reported or N/A	6 (2.4)	6 (4.4)
Infection without fever (temp <38◦C)	No dose adjustment	128 (50.4)	64 (46.4)
	Raise the daily dose slightly	104 (40.9)	48 (34.8)
	Take one extra tablet of cortisone	9 (3.5)	12 (8.7)
	At least double the daily dose	10 (3.9)	7 (5.1)
	Take one extra tablet of cortisone and call for an ambulance	0 (0.0)	0 (0.0)
	Inject Solu-Cortef and call for an ambulance	0 (0.0)	0 (0.0)
	Not reported or N/A	3 (1.2)	7 (5.1)
Infection with fever (temp >38˚C)	No dose adjustment	9 (3.5)	10 (7.3)
	Raise the daily dose slightly	62 (24.4)	34 (24.6)
	Take one extra tablet of cortisone	23 (9.1)	16 (11.6)
	At least double the daily dose	153 (60.2)	63 (45.7)
	Take one extra tablet of cortisone and call for an ambulance	3 (1.2)	6 (4.4)
	Inject Solu-Cortef and call for an ambulance	0 (0.0)	2 (1.5)
	Not reported or N/A	4 (1.6)	7 (5.1)
Occasional vomiting or diarrhoea	No dose adjustment	115 (45.3)	45 (32.6)
	Raise the daily dose slightly	47 (18.5)	23 (16.7)
	Take one extra tablet of cortisone	41 (16.1)	28 (20.3)
	At least double the daily dose	30 (11.8)	20 (14.5)
	Take one extra tablet of cortisone and call for an ambulance	11 (4.33)	10 (7.3)
	Inject Solu-Cortef and call for an ambulance	5 (2.0)	5 (3.6)
	Not reported or N/A	5 (2.0)	7 (5.1)
Recurrent vomiting or diarrhoea without fever (temp <38˚C)	No dose adjustment	34 (13.4)	15 (10.9)
	Raise the daily dose slightly	32 (12.6)	15 (10.9)
	Take one extra tablet of cortisone	18 (7.1)	11 (8.0)
	At least double the daily dose	47 (18.5)	24 (17.4)
	Take one extra tablet of cortisone and call for an ambulance	69 (27.2)	36 (26.1)
	Inject Solu-Cortef and call for an ambulance	40 (15.8)	32 (23.2)
	Not reported or N/A	14 (5.5)	5 (3.6)
Recurrent vomiting or diarrhoea with fever (temp >38˚C)	No dose adjustment	10 (3.9)	11 (8.0)
	Raise the daily dose slightly	13 (5.1)	10 (7.3)
	Take one extra tablet of cortisone	14 (5.5)	6 (4.4)
	At least double the daily dose	63 (24.8)	18 (13.0)
	Take one extra tablet of cortisone and call for an ambulance	82 (32.3)	43 (31.2)
	Inject Solu-Cortef and call for an ambulance	55 (21.7)	45 (32.6)
	Not reported or N/A	17 (6.7)	5 (3.6)
